# Glycogen synthase kinase-3β inhibition in the medial prefrontal cortex mediates paradoxical amphetamine action in a mouse model of ADHD

**DOI:** 10.3389/fnbeh.2015.00067

**Published:** 2015-03-20

**Authors:** Yi-Chun Yen, Nils C. Gassen, Andreas Zellner, Theo Rein, Rainer Landgraf, Carsten T. Wotjak, Elmira Anderzhanova

**Affiliations:** Max Planck Institute of PsychiatryMunich, Germany

**Keywords:** ADHD, mouse models, prefrontal cortex, amphetamine, NMDA receptor, dopamine, GSK3β

## Abstract

Psychostimulants show therapeutic efficacy in the treatment of attention-deficit hyperactivity disorder (ADHD). It is generally assumed that they ameliorate ADHD symptoms via interfering with monoaminergic signaling. We combined behavioral pharmacology, neurochemistry and molecular analyses to identify mechanisms underlying the paradoxical calming effect of amphetamine in low trait anxiety behavior (LAB) mice, a novel multigenetic animal model of ADHD. Amphetamine (1 mg/kg) and methylphenidate (10 mg/kg) elicited similar dopamine and norepinephrine release in the medial prefrontal cortex (mPFC) and in the striatum of LAB mice. In contrast, amphetamine decreased, while methylphenidate increased locomotor activity. This argues against changes in dopamine and/or norepinephrine release as mediators of amphetamine paradoxical effects. Instead, the calming activity of amphetamine corresponded to the inhibition of glycogen synthase kinase 3β (GSK3β) activity, specifically in the mPFC. Accordingly, not only systemic administration of the GSK3β inhibitor TDZD-8 (20 mg/kg), but also local microinjections of TDZD-8 and amphetamine into the mPFC, but not into the striatum, decreased locomotor activity in LAB mice. Amphetamine effects seem to depend on NMDA receptor signaling, since pre- or co-treatment with MK-801 (0.3 mg/kg) abolished the effects of amphetamine (1 mg/kg) on the locomotion and on the phosphorylation of GSK3β at the level of the mPFC. Taken together, the paradoxical calming effect of amphetamine in hyperactive LAB mice concurs with a decreased GSK3β activity in the mPFC. This effect appears to be independent of dopamine or norepinephrine release, but contingent on NMDA receptor signaling.

## Introduction

Attention deficit hyperactivity disorder (ADHD) is a frequent psychiatric disorder with a prevalence of up to 8% in Western populations (Faraone and Mick, 2010). It appears in three types of presentation (inattentive, hyperactive/impulsive, and combined) and shows a high degree of psychiatric comorbidity, e.g., with unipolar depression and autism spectrum disorder (Faraone et al., 2006; Piñeiro-Dieguez et al., 2014). It is generally assumed that alterations in the monoamine signaling are causally involved in the etiology of ADHD. This assumption is based on a paradoxical therapeutic efficacy of monoamine releasing drugs (amphetamine and methylphenidate and selective norepinephrine re-uptake inhibitor reboxetine) in the treatment of ADHD. Presynaptic dopamine D2 receptor-mediated downregulation of dopamine release resulting from a transient initial increase was assumed as the underlying mechanism of the paradoxical effect (Seeman and Madras, 1998).

Despite a conceivable involvement of the striatum, which mediates the dopamine-dependent locomotion, clinical evidence and effectiveness of cognitive therapy nourish the idea that the prefrontal cortex (PFC) rather than the striatum plays a critical role in ADHD pathogenesis and therapy (Mattes, 1980; Barkley, 1997). This is confirmed with recent functional imaging data showing the frontal hypoactivity in patients with ADHD (Dickstein et al., 2006). The impairment of N-methyl-D-aspartate (NMDA) receptor signaling in the medial PFC (mPFC) is believed to underlie the frontal hypoactivity and the insufficient control over vigilance and executive functions in ADHD. Disturbance in the norepinephrine and dopamine cortical neurotransmission may mediate the NMDA receptor signaling shortage (Makris et al., 2009; Arnsten and Pliszka, 2011). This suggests postsynaptic mechanisms including intracellular signaling pathways as mediators of hyperactivity and the paradoxical action of dopamine-releasing drugs.

Glycogen synthase kinase 3β (GSK3β) was primarily identified as a glycogen synthase inhibiting enzyme, a key in the glucose metabolism regulation. It also interacts with β-catenin and is intimately involved in processes of neurogenesis and neuroprotection. The non-canonical β-arrestin2/PP2A/Akt–GSK3β pathway plays a significant role in the mediation of dopamine D2/3 receptor- and amphetamine-dependent behavior. Dopamine release and activation of dopamine D2 receptors result in a decrease in the Akt activity and respective decrease in GSK3β phosphorylation leading to its activation (Beaulieu et al., 2004, 2005). Both pharmacologically-induced and innate experimental hyperactivity can be temporarily recovered by GSK3β inhibition (Emamian, 2012; Mines et al., 2013). Changes in the state of constitutionally active GSK3β were considered as both pathogenetic factor and therapeutic means in bipolar disorder and schizophrenia (Jope and Roh, 2006; Emamian, 2012). Accordingly, antipsychotics inhibit GSK3β (Emamian et al., 2004; Li et al., 2007).

GSK3β is subject to plenty of upstream regulators acting via β-arrestin2/Akt and Wnt/β-catenin, DAG/Ca^2+^-PKC, Trk R/insulin R/BDNF R-PI3K/PDK1, and PPI1-mediated pathways. Recent findings showed that not only dopamine, but also serotonin, adenosine, and brain-derived neurotrophic factor are among these upstream regulators (Jope and Roh, 2006; Kaidanovich-Beilin and Woodgett, 2011). The intracellular pathways, which can be activated by NMDA receptors, also target GSK3β (Svenningsson et al., 2003; Peineau et al., 2007; Li et al., 2009; Beurel et al., 2011; Xi et al., 2011; Liu et al., 2013).

Animal models of ADHD may help to further elucidate the biological basis of the disorder and provide deeper insight into the processes underlying effective therapeutic intervention. Most ADHD models are based on selected mutations in monoaminergic systems (Russell, 2011; Leo and Gainetdinov, 2013). However, selective breeding strategies may better resemble the multigenetic background of the disorder. We have recently validated a novel inbred ADHD mouse model, i.e., low trait anxiety-related behavior (LAB) mice (Yen et al., 2013). LAB mice were originally bred contrasting normal trait anxiety-related behavior (NAB) and high trait anxiety-related behavior (HAB) mice (Krömer et al., 2005). Selection was based on mouse exploratory behavior on the elevated plus-maze. LAB mice display a clear preference for the open arms (>60%), whereas NAB spend 20–40% and HAB <20% of the total time in the open arms. The open arm preference cannot be solely explained by low anxiety levels, but seems to reflect the increased novelty seeking (Yen et al., 2013). LAB mice of both genders show hyperactivity both in home cages (Krömer et al., 2005) and in the open field (OF; Yen et al., 2013). Remarkably, this hyperactivity becomes even stronger upon repeated exposures, thus arguing against a novelty-driven phenomenon (Yen et al., 2013). LAB mouse endophenotype is characterized by an increase in acoustic startle responses, impaired social recognition and spatial memory. Compared to HAB mice, LAB mice display changes in metabolic pathways both in the periphery and in the brain (Krömer et al., 2005; Kessler et al., 2007; Filiou et al., 2011). In terms of predictive validity, LAB mice show a paradoxical calming response to amphetamine in non-toxic doses (0.5–2.0 mg/kg, i.p.) (Yen et al., 2013).

The aim of the present study was to elucidate the neurochemical and molecular basis of the paradoxical effect of amphetamine in our mouse model of ADHD (i.e., LAB mice) with a special focus on the role of GSK3β. To this end, (1) we examined locomotor activity in the OF and employed *in vivo* microdialysis in order to compare behavioral effects of amphetamine (and methylphenidate) in LAB and HAB mice with drug-related changes in the dopamine and norepinephrine levels in the mPFC and the striatum. We assessed (2) the efficiency of the dopamine D2 receptor function in LAB mice by measuring behavioral and neurochemical effects of haloperidol. We explored (3) the effects of amphetamine treatment on the phosphorylation of GSK3β in the two brain structures under study; and (4) the effects of systemic and local inhibition of GSK3β on locomotor activity. Finally, we examined (5) potential involvement of glutamate signaling via NMDA receptors in the mechanisms of the calming effect of amphetamine.

## Material and Methods

### Animals

Male HAB, LAB and normal trait anxiety-related behavior (NAB) mice are selectively bred from Swiss CD1 mice (Charles River, Sulzfeld, Germany) in the Max Planck Institute of Psychiatry. Hyperactivity of LAB mice is observed in single- and group-housed animals during light and dark phases of the diurnal cycle and appears in two types: (i) LAB-Intermediate (LAB-I) mice are animals with non-habituating locomotion slightly exceeding the ambient activity in NAB and HAB mice; and (ii) LAB-Strong (LAB-S) mice showing 3-fold higher locomotor activity (Yen et al., 2013). All experiments presented here were performed in LAB-I mice, which represent the majority of the offspring (>60%) and most closely fulfill the criteria for an animal model of ADHD (Yen et al., 2013). For the sake of clarity, the abbreviation “LAB” is used instead of “LAB-I” throughout the manuscript. All mice were single-housed under standard laboratory conditions with reversed 12/12 h light/dark cycle (light on at 9 pm), temperature 23 ± 1°C, food and water ad libitum approximately 2 weeks before experiments started. We performed basal locomotor tests with all mice in order to exclude LAB-strong animals, followed by verification of the calming response of amphetamine at an age of 2.0–2.5 months. To meet the 3R’s rule of animal welfare we repeatedly (3–4 times) tested mice that was possible due to a stability of endophenotypes (Yen et al., 2013). Given the inter-trial intervals (5–10 days) animals were available for subsequent tests at an age of 3–6 months. Nonetheless, to exclude any confounding effect of the repeated exposure animal to the OF and/or a carryover effect of injections and previous treatments, we always analyzed the basal activity measured before any treatment (first 20 min of the OF test). Experiments with between-line comparisons were performed at the same time. Number of animals in the experimental groups varied from 4–13. The exact sample size is indicated in the figures/figure legends. All experiments were carried out according to the European Community Council Directive 2010/63/EEC, and efforts were made to minimize animal suffering. All experimental procedures were approved by the local government of Upper Bavaria (55.2.1.54-2532-188-12).

### Drugs and Doses

d-Amphetamine hemisulfate (Amph), methylphenidate hydrochloride, lithium chloride (LiCl), TDZD-8, and dimethyl sulfoxide (DMSO) were from Sigma-Aldrich (USA). Haloperidol stock solution was from Ratiopharm GmbH (Germany). Amphetamine, methylphenidate, LiCl powders and haloperidol stock solution were dissolved in saline. TDZD-8 stock solution was prepared in 100% DMSO and then dissolved in saline to achieve a final DMSO concentration of 0.5% v/v. All working solutions were prepared freshly before each experiment. Amphetamine (0.5, 1, and 2 mg/kg) and methylphenidate (3, 10, and 30 mg/kg) were injected in doses which did not induce toxic effect and result in stereotypic behavior. Haloperidol was injected at the dose of 1 mg/kg for reproducing the catalepsy (Boulay et al., 2000; McOmish et al., 2012). The LiCl dose (100 mg/kg) was chosen to trade-off the specific antimanic and gustatory/digestive tract aversive effects of this drug (Gould et al., 2007). The TDZD-8 dose (20 mg/kg) was selected on the basis of our preliminary experiments (not shown) and the previous report (Beaulieu et al., 2004). All drugs were injected intraperitoneally (i.p.) in the volume of 100 µl/10 g of body weight. The doses for amphetamine (1.8 ng/0.5 µl/side) and TDZD-8 (1.1 ng/0.5 µl/side) microinjections were chosen on the basis of the previous reports (Prasad et al., 1999; Chen et al., 2004; Ramirez et al., 2010). Control groups received the respective vehicle injection/microinjection, saline or 0.5% DMSO.

### Behavioral Tests

All behavioral experiments were performed during the active phase of the diurnal cycle between 10 am and 6 pm.

**Locomotor activity** was assessed in the OF test by measurement of the distance traveled with the automatic TruScan Photo Beam Activity system (Coulbourn Instruments, Whitehall, PA, USA) as described previously (Yen et al., 2013). Basal activity was measured within 20 min prior to systemic drug administration. After an i.p. injection (which lasted less a 1 min) mice were returned to the test arena and recording was continued for 1 or 2 h. In the case of mPFC and striatum local treatment, recording started 2–3 min after microinjections and lasted for 1 h. Data were analyzed in 5 min bins; in some cases, we report mean data corresponding to either 20 min intervals or the entire observation period.

**Stereotypic behavior assessment** was done in accordance to Havemann et al. (1986) rating scale: 0 (no stereotypies); 1 (discontinuous sniffing); 2(continuous sniffing); 3 (discontinuous licking); 4 (continuous licking); 5 (discontinuous gnawing); 6 (continuous gnawing).

**Catalepsy test** was performed as described (Sanberg, 1980). Modification was done by adjusting the horizontal bar in a way that the mouse’s forepaws were paced 3 cm above the floor level. The latency of the mouse to descend from the inconvenient postures was recorded by a trained observer; immobility for more than 5 min was scored as 300 s.

### Brain Microdialysis

**Surgery, probe implantation, and microdialysis** were done as described before (Anderzhanova et al., 2013). Microdialysis guide cannulas (Microbiotech/se AB, Sweden) were implanted into the right mPFC (coordinates: AP 2.20 mm, ML 0.35 mm, and DV −1.50 mm) or right striatum (coordinates: AP 0.50 mm, ML 2.00 mm, and DV −2.25 mm) in accordance with Paxinos and Franklin Mouse Brain Atlas (Paxinos and Franklin, 2001) under isoflurane (Abbot, India) Metacam® (Boehringer Ingelheim GmbH, Germany) anesthesia. Recovery lasted for 1 week and included Metacam® supplementation 0.25 mg/100 ml with drinking water. Microdialysis probes (o.d. 0.2 mm, cuprophane membrane 2 mm of length, MAB 4.15.2.Cu, Microbiotech/se AB, Sweden) were inserted under slight isoflurane anesthesia and then continuously perfused with sterile artificial cerebrospinal fluid (concentrations, in mM: NaCl 145, KCl 2.7, CaCl_2_ 1.2, MgCl_2_ 1.0, Na_2_HPO_4_ 2.0, pH = 7.4). Microdialysis fractions (20 min) were collected during experimental days 1, 2 and 3 at a flow rate of 1.0 µl/min. In order to minimize the number of animals, microdialysis experiments lasted for three consecutive days during which different pharmacological treatments were given in the same order for each animal. Amphetamine and methylphenidate were injected on the first day 5 h apart allowing recovery of both behavior and catecholamine levels. Amphetamine and MK-801 or saline were injected on the second day, haloperidol on the third day.

**Monoamine assays**. Dopamine, norepinephrine, and homovanillic acid (HVA) contents were determined by reverse-phase HPLC with electrochemical detection (UltiMate3000 CoulochemIII, ThermoFischer, USA). All reagents used for the mobile phase were of analytical grade (Carl Roth GmbH or MERCK KGaA, Germany). Monoamines were separated on an analytical column (C18, 150 mm × 3 mm, 3 µm, YMC Triart, YMC Europe GmbH, Germany) at a flow rate of 0.4 ml/min. The potentials of the working electrodes were set at −150 mV, +220 mV, the guard cell potential was set at +350 mV. Monoamine concentrations were calculated by external standard curve calibration using the peak area for quantification. The detection limits for norepinephrine and dopamine were 0.032 and 0.040 nM, respectively; therefore few data sets were excluded from analysis.

Basal levels were not corrected by an *in vitro* recovery examination. The values were stable across 3 days of measurement both in the mPFC and in the striatum. One-way ANOVA did not reveal any differences between the day groups in HAB or LAB mice (*Fs* < 0.74, *ps* > 0.40).

### Drug Microinjections

Custom-designed stainless steel injection cannulas (23G) were bilaterally implanted into the mPFC (AP 1.90 mm, ML ± 0.40 mm, and DV −2.00 mm; Figure 5A) and the striatum (AP 0.50 mm, ML ± 2.00 mm, and DV −3.00 mm; Figure 5D) (Paxinos and Franklin, 2001). Recovery took 1–2 weeks. Injections were done directly before OF tests under slight isoflurane anesthesia. Injections were performed by means of a cannula (0.3 mm o.d.), which was connected to a microliter syringe (65RNR 10.0 µL SYR, Hamilton Bonaduz AG, Switzerland) via calibrated tubing. Once inserted, the injection cannula protruded from the guide cannula1 mm, thus reaching the prelimbic mPFC or the dorso-lateral striatum. Different injection sets were used for drug and vehicle.

**Figure 1:**
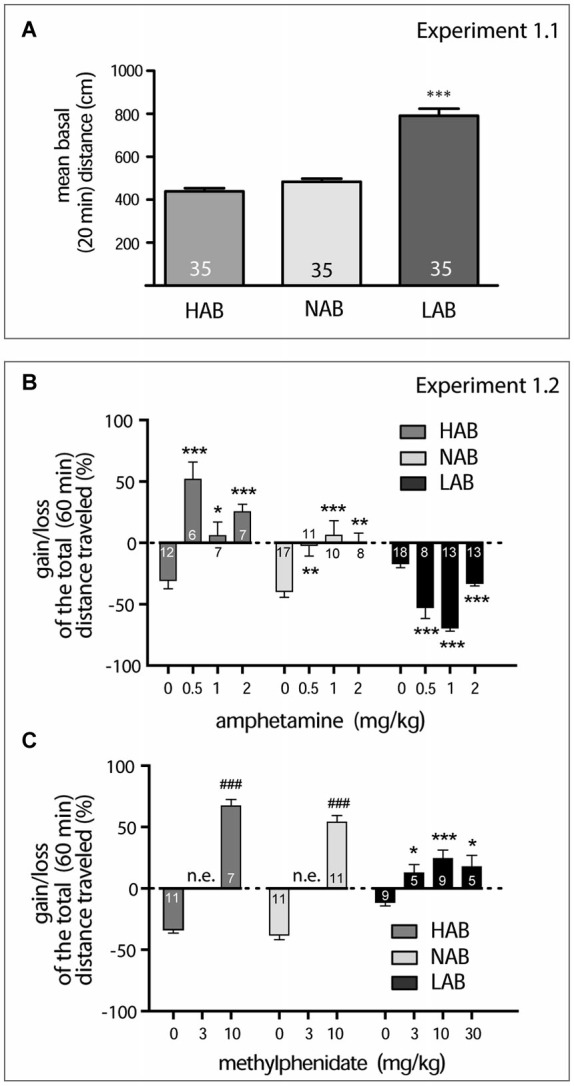
**Amphetamine and methylphenidate action on locomotor activity (A)**. LAB mice are strictly different from NAB and HAB mice in their mean basal locomotor activity observed within 20 min in the OF (***p < 0.001). **(B)**. Dose-dependent effect of amphetamine on locomotor activity in OF test. Here and in the next graph, data represent relative changes (gain or loss) in the mean total distance traveled within 60 min after drug administration in comparison to basal activities (last 5 min of pre-treatment 20 min period). Asterisks show the result of the Dunnett’s *post hoc* test comparing the effect of different doses with saline effect. **(C)**. Dose-dependent effect of methylphenidate on locomotor activity in OF test. The two-tailed Student’s test in the cases of HAB and NAB mice revealed a difference (depicted with hash marks, ^###^*p* < 0.001) between changes in locomotor activity after saline and drug treatment. (Asterisks show the result of the Dunnett’s *post hoc* test comparing the effect of different doses with saline effect in LAB mice. *p < 0.05, **p < 0.01, ***p < 0.001; n.e. not examined; numbers on the graph panels represent group size.

**Figure 2:**
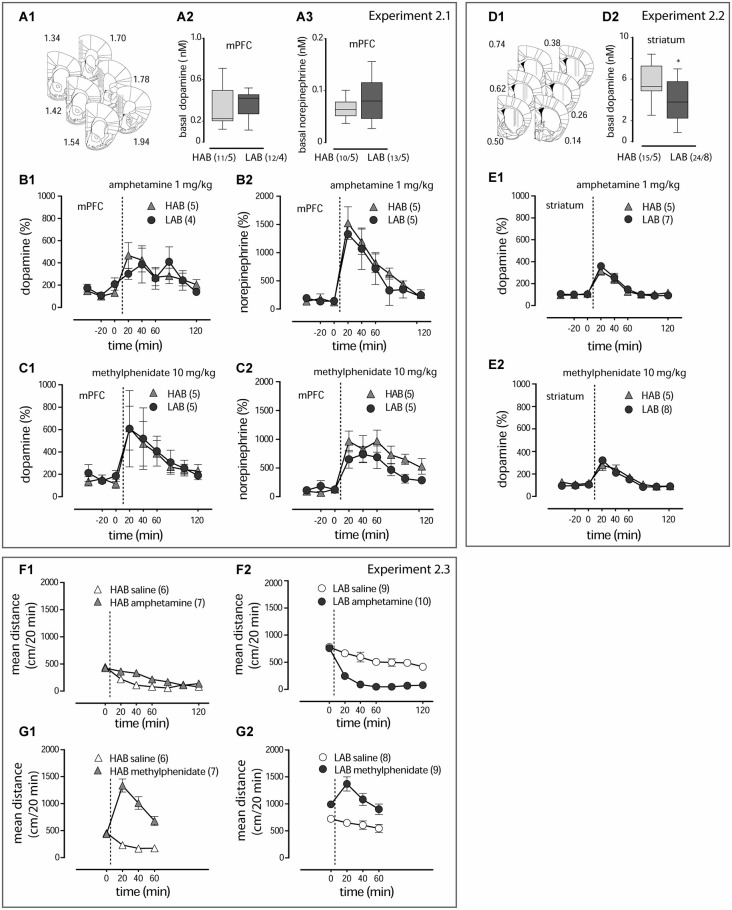
**Amphetamine and methylphenidate action on dopamine and norepinephrine release (A1, D1)**. The schematic diagrams show placement of microdialysis probes in subsequent coronal sections of the mPFC and striatum. The computer-based atlas by Paxinos and Franklin (2001) was used to mark probe locations; numbers refer to distances from the bregma, mm. **(A2,3,D2)**. Basal values of dopamine and norepinephrine in the mPFC and dopamine in the striatum of LAB and HAB mice. *p < 0.01; “n/N” represent the sample size/number of animals under evaluation. Amphetamine (1 mg/kg, i.p.) and methylphenidate (10 mg/kg, i.p.) evoke comparable dopamine and norepinephrine release in the mPFC **(B1,2,C1,2**) and striatum **(E1,2**) of both LAB (circles) and HAB (triangles) mice. Amphetamine (1 mg/kg, i.p.) decreases hyperactivity in LAB mice (circles in contrast to the stimulatory effect in HAB mice (triangles) **(F1,2)**. Methylphenidate (10 mg/kg, i.p.) stimulates locomotion in both lines **(G1,2)**. The dashed lines define the moment of drug administration.

**Figure 3:**
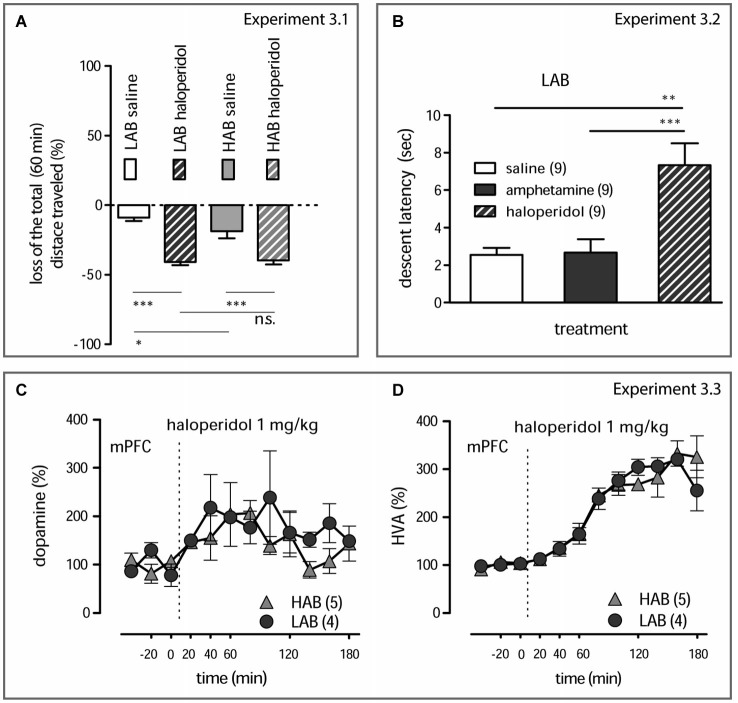
**Behavioral and neurochemical effects of haloperidol (A)**. Haloperidol (1 mg/kg, i.p.) decreases the total distance traveled by both LAB and HAB mice; the corresponding dynamical data are presented in Yen et al. (2013). Relative gains or losses in the mean distance traveled within 60 min after treatment was obtained in comparison to basal activities (last 5 min of pre-treatment 20 min period). A subtle difference between saline-treated LAB and HAB mice reflecting the already reported difference in the short-term habituation rate in these animals (Yen et al., 2013). However, the locomotion-suppressive effects of haloperidol (1 mg/kg, i.p.) were prominent both in LAB and HAB mice and did not differ between lines.***p < 0.001, *p < 0.05, n.s. non significant (Newman-Keuls’s *post hoc* comparison). **(B)**. Mitigation of locomotor activity by haloperidol (1 mg/kg, i.p.) in LAB mice is mediated by its characteristic neuroleptic activity (development of a cataleptic state) which can be discriminated from the calming action of amphetamine (1 mg/kg, i.p.). **p < 0.01, ***p < 0.001 (Neumann-Keuls’s *post hoc* comparison). **(C,D)**. Haloperidol (1 mg/kg, i.p.) increases dopamine release in the mPFC and evokes equal accumulation of HVA in both LAB (circles) and HAB (triangles) mice. The dashed line defines the moment of drug administration.

**Figure 4:**
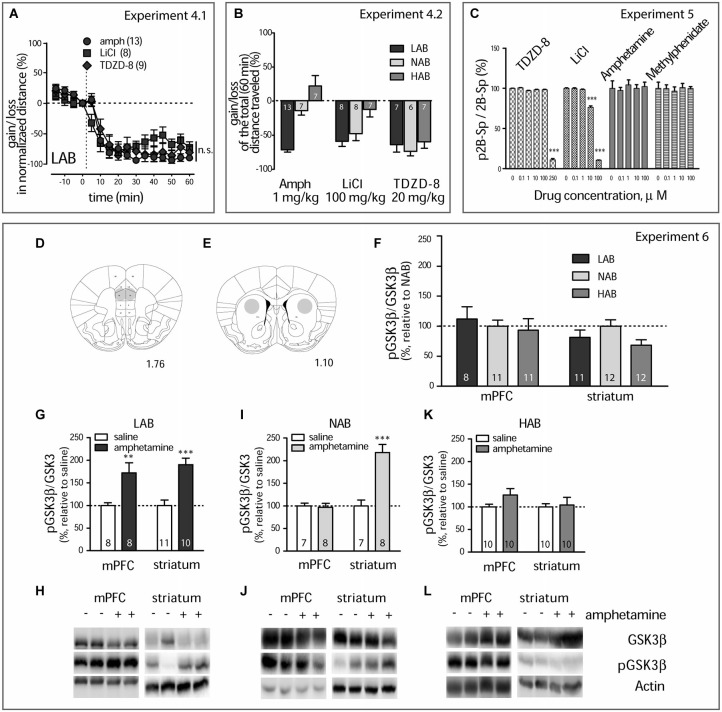
**Amphetamine treatment results in GSK3β inhibition (A)**. Amphetamine (1 mg/kg, i.p., circles), LiCl (100 mg/kg, i.p., squares), and TDZD-8 (20 mg/kg, i.p., diamonds) mitigate the locomotor activity in LAB mice with the same dynamics. The dashed line defines the moment of drug injection; n.s. non significant. **(B)**. Within-line comparison of amphetamine (1 mg/kg, i.p.), LiCl (100 mg/kg, i.p.), TDZD-8 (20 mg/kg, i.p.), and saline effects on the locomotor activity in LAB, NAB, and HAB mice illustrates the similar ability of all drugs to decrease the traveled distance in LAB mice. Relative gains or losses in the mean distance traveled within 60 min after treatment was obtained in comparison to basal activities (last 5 min of pre-treatment 20 min period). Analysis confirms the selectivity of amphetamine action in LAB mice and points to a lack in line specify of LiCl and, in particular, TDZD-8 to inhibit locomotion. *p < 0.05, **p < 0.01, ***p < 0.001, within line comparison, Bonferroni’s *post hoc* test. Numbers on the bars represent the group size. The same order of treatments was applied for each line. **(C)**. Lithium chloride and TDZD-8 show a direct inhibition of recombinant GSK3β *in vitro* effectively reducing (*p* < 0.001) its activity in a dose-dependent manner. Neither amphetamine nor methylphenidate inhibit recombinant GSK3β *in vitro*. **(D,E)**. Schematic representation of punched areas in the mPFC and the striatum. The computer-based atlas by Paxinos and Franklin (2001) was used to mark probe locations; numbers refer to distances from the bregma, mm. **(F)**. Western blot kinase analysis for the mPFC and the striatum of drug naïve mice shows no difference between LAB (*n* = 8/11), NAB (*n* = 11/12) and HAB mice (*n* = 11/12) in pGSK3β levels. **(G,I,K)**. Changes in the pGSK3β levels in the mPFC and the striatum of LAB, NAB, and HAB mice 60 min after amphetamine (1 mg/kg, i.p.) and saline treatment and coinciding with OF exposure. **p < 0.01, ***p < 0.001 vs. saline **(H,J,L)**. Representative Western blots for analysis of GSK3β, pGSK3β protein level. Bands include samples both from amphetamine and saline-treated animals. Numbers on the graph panels represent group size.

**Figure 5:**
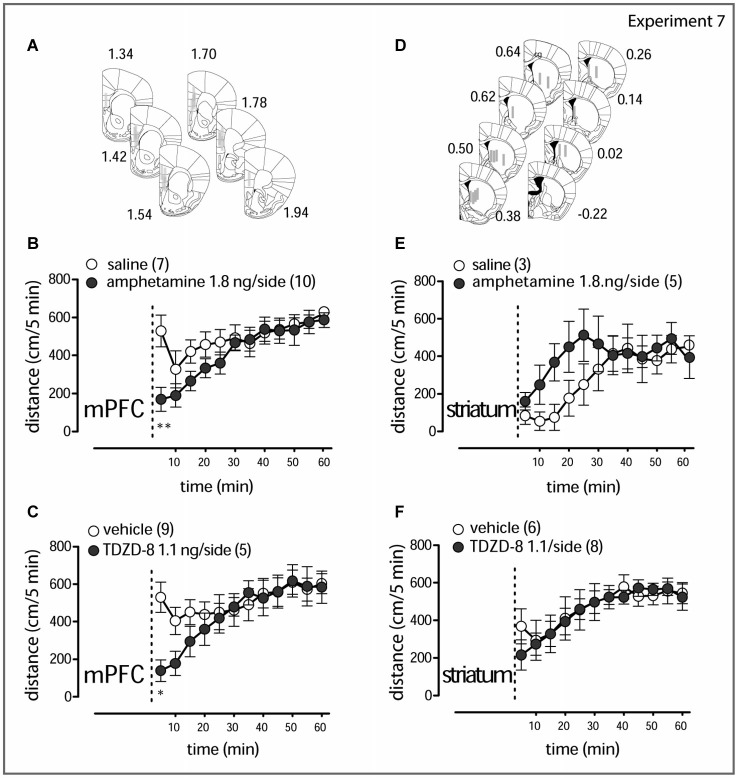
**Local amphetamine and TDZD-8 treatment (A,D)**. Schematic representation of the drug infusion loci. The rectangles correspond to tracks of the injection cannula tip (1 mm) in the mPFC and the striatum. Results for right and left hemisphere examinations are pooled together. The computer-based atlas by Paxinos and Franklin (2001) was used to mark probe locations; numbers refer to distances from the bregma, mm. Amphetamine (1.8 ng/side, **B**) and TDZD-8 (1.1 ng/side, **C**) transiently counteract the hyperlocomotor activity in LAB mice when injected in the mPFC. Amphetamine (1.8 ng/side, **E**) rather stimulates locomotion, when injected in the striatum, whereas TDZD-8 (1.1 ng/side, **F**) does not exert any action. The dashed lines mark the moment of drug administration (2–3 min before the animal is placed into the OF test box). Open circles correspond to saline/vehicle treatment; filled circles correspond to drug treatment. *p < 0.05, **p < 0.01.

### Tissue Isolation from Frozen Brains

Animals were i.p. injected with saline, amphetamine (1 mg/kg), and MK-801 (0.3 mg/kg) in accordance with the corresponding protocols and examined in the OF test. Sixty minutes after the injections, animals were lightly anesthetized and decapitated, brains removed, immediately frozen on dry ice, and kept at −80°C. Biopsy punches of the mPFC and the striatum were done with pre-chilled stainless steel sample corers (diameters of 0.8 mm and 1.0 mm for the mPFC and striatum, respectively) (Fine Science Tools GmbH, Germany) from coronal sections of the brains at −20°C (Tzigaret et al., 1993). The withdrawn regions are schematically depicted in Figures 4D,E. Specimens from the right and left hemispheres were pooled together and stored at −80°C prior to western blot analysis.

### Western Blot Analysis

Western blot analysis was performed as described previously (Zschocke et al., 2011). Protein extracts were obtained by lysing the brain punches in 62.5 mM Tris, 2% SDS and 10% sucrose, completed with protease (Sigma, P2714) and phosphatase (Roche, 04906837001) inhibitor cocktail. Samples were sonicated and heated at 95°C for 10 min. SDS-PAGE was carried out to separate proteins. Proteins were electro-transferred onto nitrocellulose membranes. Blots were placed in Tris-buffered saline (TBS), supplemented with 0.05% Tween (Sigma, P2287, USA) and 5% non-fat milk for 1 h at room temperature and then incubated with primary antibody (diluted in TBS/0.05% Tween) overnight at 4°C. The following primary antibodies were used: GSK3β (1:1000, Cell Signaling, #9315), phospho-Ser9-GSK3β (1:1000, Cell Signaling, #9323), Actin (1:5000, Santa Cruz Biotechnologies, sc-1616), HSC70 (1:5000, Santa Cruz Biotechnologies, sc-7298). Subsequently, blots were washed and probed with the respective horseradish peroxidase- or fluorophore-conjugated secondary antibody for 1 h at room temperature. The immuno-reactive bands were visualized either by using ECL detection reagent (Millipore, Billerica, MA, USA, WBKL0500) or directly by excitation of the respective fluorophore. Determination of the band intensities was performed with ChemiDoc MP (BioRad, CA, USA). The Western blot protocols were adapted to process small quantities of biological material which could be obtained upon brain structure punching. Low content of proteins and respective adjustments of the Western blot image reader sensitivity in particular cases influenced the image quality. Nonetheless, the sample bits were enough for reliable quantification. Comparison for optical density revealed a difference between the experimental groups.

The phosphorylation index was used as an indicator of kinase activity. Drug and line effects were evaluated in comparison to either saline- (Figures 4G,I,K) and amphetamine- (Figure 7D) treated groups or NAB mice (Figure 4F). Drug-induced dose-dependent changes were evaluated after normalization to vehicle-treated specimens.

**Figure 6:**
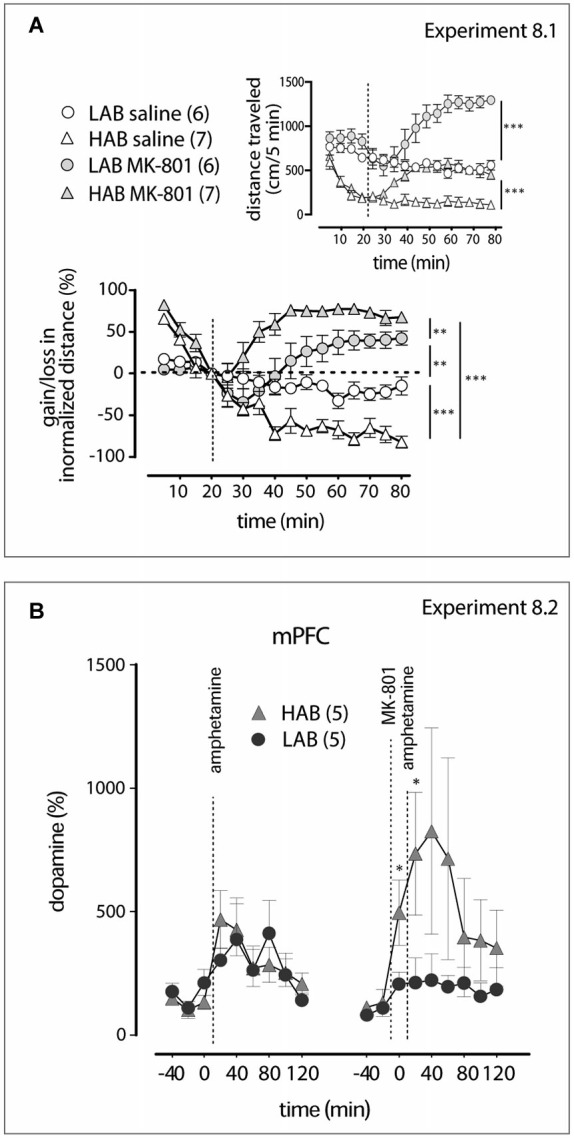
**MK-801 shows different activities in LAB and HAB mice (A)**. Relative increase in locomotor activity induced by the NMDA receptor antagonist MK-801 is more pronounced in HAB (triangles) than in LAB (circles) mice. The dashed lines mark the moments of saline or MK-801 (0.3 mg/kg, i.p.) administration. The inset graph represents changes in the absolute values of the distance traveled. **p < 0.01, ***p < 0.001). **(B)**. As revealed by *in vivo* microdialysis, pre-treatment (−20 min) with MK-801 facilitates dopamine release in the mPFC of HAB (triangles), but not of LAB (circle), mice. The dashed lines mark the moments of amphetamine (1 mg/kg, i.p.) and MK-801 (0.3 mg/kg, i.p.) administrations. *p < 0.05 vs. LAB mice.

**Figure 7:**
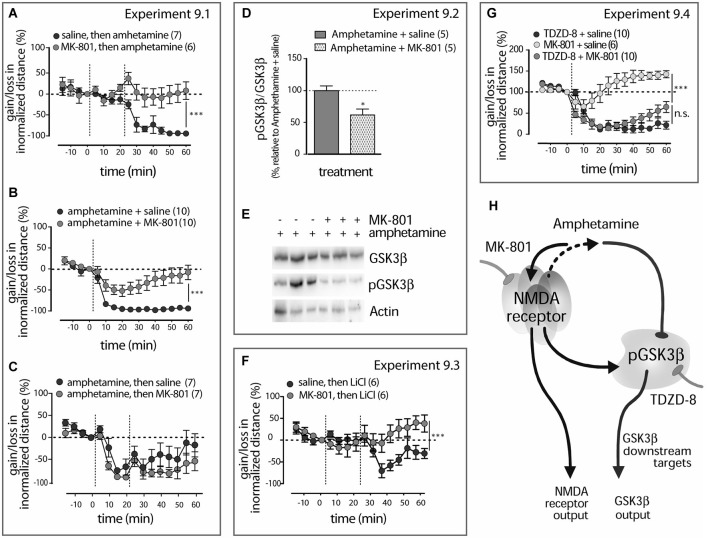
**Amphetamine action in LAB mice interfere with NMDA receptor signaling (A)**. Pre-treatment (−20 min) with MK-801 abolishes amphetamine effect on the locomotor activity in LAB mice. The dashed lines mark the moments either of saline and amphetamine (1 mg/kg, i.p.) or MK-801 (0.3 mg/kg, i.p.) and amphetamine (1 mg/kg, i.p.) administration. **p < 0.01. **(B)**. Co-treatment with MK-801 counteracts the amphetamine calming effect in LAB mice. The dashed lines mark the moments of either saline + amphetamine (1 mg/kg, i.p.) or MK-801 (0.3 mg/kg, i.p.) + amphetamine (1 mg/kg, i.p.) administration. ***p < 0.001. **(C)**. MK-801 post-treatment (+20 min) did not interfere with amphetamine action. The dashed lines mark the moments of amphetamine (1 mg/kg, i.p.) and MK-801 (0.3 mg/kg, i.p.) injections. **(D)**. Decrease in the phospho-Ser9-GSK3β levels in the mPFC of LAB (*n* = 5/5) mice 60 min after MK-801 (0.3 mg/kg, i.p) and amphetamine (1 mg/kg, i.p.) co-treatment and coinciding with OF exposure. *p < 0.05 vs. amphetamine + saline. **(E)**. Representative Western blots for the analysis of GSK3β, pGSK3β protein levels in LAB mice. Bands include samples both from amphetamine + saline- and amphetamine + MK-801-treated animals. **(F)**. MK-801 pre-treatment (−20 min) effectively collapses the calming effect of LiCl. The dashed lines mark the moments of MK-801 (0.3 mg/kg, i.p.) and LiCl (100 mg/kg, i.p.) injections. ***p < 0.0001. **(G)**. MK-801 does not interfere with the activity of the pure GSK3β inhibitor TDZD-8 in its ability to modulate locomotor activity in LAB mice. The dashed lines mark the moments of either MK-801 (0.3 mg/kg), saline + TDZD-8 (20 mg/kg, i.p.) or MK-801 (0.3 mg/kg, i.p.) + TDZD-8 (20 mg/kg, i.p.) administration. Since there was no difference between saline and 0.5% DMSO treatment, results for both vehicle-treated groups were pooled together. ***p < 0.001, n.s. non significant. **(H)**. Hypothetical scenario: amphetamine effects on GSK3β comprise NMDA receptor-mediated kinase slowdown (due to glutamate release) rather than any direct effect. This pathway, unidentified in details, is uniquely activated in the mPFC of LAB mice and leads to GSK3β inhibition. Hypofunction of NMDA receptors might be a permissive cause of this pathway activation.

### Mass Spectrometry-Based GSK3β Kinase Assay

Determination of GSK3β kinase activity in the presence of TDZD-8, LiCl, methylphenidate, amphetamine, or vehicle was done as in Bowley et al. (2005) with slight modifications. The kinase reaction buffer was supplemented with 1 mM ATP, 0.2 mM DTT, protease inhibitor (Sigma, P2714), phosphatase inhibitor (Roche, 04906837001), and 45 ng of recombinant GSK3β (BPS Bioscience, CA, USA). A MALDI-ToF mass spectrometer (Ultraflex I, Bruker Daltronics) was used for analysis. The ratio between concentrations of phosphorylated and total substrate 2B-Sp was used as an index of kinase activity.

### Histology

Cryo-sections of 25 µm obtained from target or punched brain regions were stained with cresyl violet (Carl Roth GmbH, Germany) and verified under a microscope using the Paxinos and Franklin mouse atlas (Paxinos and Franklin, 2001). Schematic representations of the targeted regions are shown in Figures 2A,D, 4D,E, 5A,D. When probe/cannula placement was found to be out of the targeting area, the respective samples/animals were discarded before analysis.

#### Experiments’ Outline

Different cohorts of LAB, NAB and HAB mice were used in each experiment. The sizes of groups are indicated in the Figures.

**Experiment 1.1**. Comparison of the mean basal locomotor activities (20 min OF test in drug naïve animals) between LAB, NAB, and HAB mice. **Experiment 1.2**. Between line (HAB, NAB and LAB mice) comparison of amphetamine (0.5, 1.0, and 2.0 mg/kg, i.p) and methylphenidate (3, 10, 30 mg/kg, i.p.) dose-response effects on the gain or loss in the mean locomotor activity within 60 min after drug administration (OF test).

**Experiment 2.1**. Evaluation of acute neurochemical changes in the mPFC of LAB and HAB mice evoked by systemic administration of amphetamine (1 mg/kg, i.p.; this dose is used in the next experiments) and methylphenidate (10 mg/kg) (microdialysis). **Experiment 2.2**. Evaluation of acute neurochemical changes in the striatum of LAB and HAB mice evoked by systemic administration of amphetamine and methylphenidate (microdialysis). **Experiment 2.3**. Examination of the locomotor activity dynamics within 60–120 min after systemic amphetamine and methylphenidate treatment (OF test).

**Experiment 3.1**. Comparison of haloperidol (1 mg/kg, i.p.) effects on the loss in the mean locomotor activity within 60 min after drug administration in LAB and HAB mice (OF test). **Experiment 3.2**. Examination of cataleptogenic activities of amphetamine and haloperidol in LAB mice (descent latency measurement at 60 min after drug administration). **Experiment 3.3**. Evaluation of acute neurochemical changes in the mPFC after haloperidol treatment in LAB and HAB mice (microdialysis).

**Experiment 4.1**. Comparison of the locomotor activity within 60 min after GSK3β inhibitors (TDZD-8, 20 mg/kg and LiCl, 100 mg/kg, i.p; these doses are used in the next experiments) and amphetamine administration in LAB mice (OF test). **Experiment 4.2**. Comparison of TDZD-8, LiCl, and amphetamine effects on the loss or gain in the mean locomotor activity within 60 min after drug administration in LAB, NAB, and HAB mice (OF test).

**Experiment 5**. Comparison of the amphetamine, methylphenidate, LiCl, and TDZD-8 GSK3β inhibitory activity *in vitro*.

**Experiment 6**. Examination of systemic amphetamine administration effects on GSK3β phosphorylation levels observed at 60 min after drug i.p. injection in the mPFC and in the striatum of LAB, NAB, and HAB mice (Western blot).

**Experiment 7**. Comparison of changes in locomotor activity within 60 min after amphetamine and TDZD-8 bilateral microinjections into the mPFC and into the striatum in LAB mice (OF test).

**Experiment 8.1**. Evaluation of the locomotor activity dynamics within 60 min after MK-801 (0.3 mg/kg, i.p.; this dose is used in the next experiments) treatment in LAB and HAB mice (OF test). **Experiment 8.2**. Examination of MK-801 effects of the amphetamine-induced dopamine release in the mPFC of LAB and HAB mice (microdialysis).

**Experiment 9.1**. Comparison of administration time-dependent (pre-, co- and post-treatment) effects MK-801 on the amphetamine-mediated locomotion mitigation in LAB mice (OF test). **Experiment 9.2**. Evaluation of the MK-801 and amphetamine interaction in regulation of GSK3β phosphorylation in the mPFC of LAB mice (Western blot). **Experiment 9.3**. Examination of MK-801 pre-treatment effects on the LiCl-evoked locomotion mitigation in LAB mice (OF test). **Experiment 9.4**. Examination of MK-801 co-treatment effects on TDZD-8-evoked locomotion mitigation in LAB mice (OF test).

#### Data and Statistical Analysis

Considering the basal difference in the absolute values of locomotor activities between LAB and HAB mice (Figures 1A, 2F,F,G,G), the OF data were normalized to better visualize relative changes in the traveled distance after drug administration. The applied algorithm (Hinkelmann et al., 2010) employs a comparison of running values (*x*_i_) and measurement of the last 5 min of the pretreatment period (*x*_4_). Relative changes were calculated in accordance with the equation: $x_{\text{i}}\left(\%\right)=200\cdot \left(x_{\text{i}}^{2}\right)/\left(x_{\text{i}}^{2}+x_{4}^{2}\right)$. Gains and losses in the distance traveled were obtained by subtraction of normalized values from 100%. Such a way of data normalization ensures that relative changes in locomotion will be displayed in the ranges of 0%+100% (gain) and 0%−100% (loss), thus avoiding any bias towards increase locomotion on expenses of decreased locomotion. Microdialysis data were expressed as a percentage of absolute dopamine and norepinephrine basal values (basal values were the means of three consecutive samples) or as absolute concentrations in the microdialysates (for basal levels only). All data are expressed as mean ± S.E.M. The sample sizes were chosen on the basis of our previous experience with the procedures used, and they are adequate to detect meaningful differences between conditions. Statistical analyses were performed with Statistica, version 5.0 (StatSoft Inc., Tusla, OK, USA). Data were analyzed with the Kolmogorov-Smirnov for distribution and met the assumptions of the test with regard to normality, skew and homogeneity of variance. Mice of each line were randomly assigned to the treatments for between-groups comparison in microdialysis experiments. Counterbalanced assignment of treatment order for within-subject design was used in behavioral experiments. Experimenters were blind to either the subline or the treatment assignments. Statistics were two-tailed *t*-test, one-way ANOVA, two-way ANOVA (the factorial design included the time and line or treatment factors for analysis of dynamic locomotor activity and microdialysis data; structure and treatment factors for analysis for molecular data; in any other cases the design is specified particularly in the respective part of Results) and followed by Neuman-Keuls’s or Dunnett’s or Bonferoni’s *post hoc* tests, if appropriate. All differences were considered significant at *p* < 0.05.

## Results

### The Calming Effect of Amphetamine is not Mirrored by Changes in Monoamine Release

To confirm the hyperactive phenotype of LAB mice (Yen et al., 2013), we exposed LAB, NAB and HAB mice to an OF for 20 min (Experiment 1.1). LAB mice showed the elevated mean basal activity during 20 min exposure to the OF (791.0 ± 33.12 cm) compared to NAB (484.0 ± 13.66 cm) and HAB mice (438.7 ± 15.21 cm), which were indistinguishable from each other (one-way ANOVA with Bonferroni’s *post hoc* test; *F*_(2)_ = 72.72, *p* < 0.0001; Figure 1A).

LAB mice differed from NAB and HAB mice in their profile of changes in the mean total distance traveled after amphetamine and methylphenidate administration (Experiment 1.2). Amphetamine exerted a calming activity in LAB mice and increased locomotion in NAB and HAB mice in a range of doses (0.5–2.0 mg/kg, i.p.). Two-way ANOVA (line, dose) exploring amphetamine effects showed significance for all factors (line: *F*_(2,130)_ = 61.97, *p* < 0.0001; dose: *F*_(3,130)_ = 12.03, *p* < 0.0001; line x dose: *F*_(6,130)_ = 19.79, *p* < 0.0001). The subsequent one-way ANOVAs performed separately per line points to significance of amphetamine dose effect in each line (LAB: *F*_(3)_ = 45.71, *p* < 0.001; NAB: *F*_(3)_ = 8.87, *p* = 0.001; HAB: *F*_(3)_ = 15.46, *p* < 0.001; Figure 1B), however expressed in a different directions. Methylphenidate showed its stimulatory activity in all lines injected with 3 doses (3.0, 10.0, 30.0 mg/kg). The one-way ANOVA for LAB mice points to significant dose effect (*F*_(3)_ = 7.20, *p* = 0.0013). The two-tailed Student’s test in the cases of HAB and NAB mice revealed a difference between changes in locomotor activity after saline and drug treatment (HAB mice, *t*_(16)_ = 18.05, *p* < 0.007, NAB mice *t*_(20)_ = 13.72, *p* < 0.0001; Figure 1C). The stereotypia scores were always of zero levels. In no case did we detect even initial signs of amphetamine-specific (discontinuous sniffing) stereotypic behavior which might have explained the decrease in locomotor activity found in LAB mice.

On the basis of these results and considering the monoamine releasing potency of amphetamine and methylphenidate reported in the literature, we selected 1 mg/kg for amphetamine and 10 mg/kg for methylphenidate for the subsequent neurochemical studies. In these studies, we asked whether the differences in behavioral effects of amphetamine vs. methylphenidate in LAB mice (i.e., reduction vs. increase in locomotor activity) were reflected by similar differences in dopamine and norepinephrine levels in the mPFC and/or the striatum. This was done in comparison to HAB mice, which were most responsive to any of the drugs in terms of increased locomotion. Microdialysis was performed in the mPFC and in the striatum in independent groups of mice.

In the first cohort of animals (Experiment 2.1) microdialysis probes targeted the mPFC including the cingulate cortex (Cg1), the prelimbic cortex (PrL) and the infralimbic cortex (IL; Figure 2A). Mean basal catecholamine levels were evaluated in the entire pool of basal samples in drug-naive animals (day 1) (HAB, *n* = 5; LAB, *n* = 5). Comparison of the absolute extracellular dopamine levels measured in the mPFC failed to reveal any line differences for both the basal dopamine (*t*_(21)_ = 0.61, *p* = 0.556; Figure 2A) and the norepinephrine (*t*_(21)_ = 1.22, *p* = 0.236; Figure 2A) content.

As revealed by two-way ANOVAs (line, time), both amphetamine (1 mg/kg, i.p.) (time: *F*_(8,66)_ = 3.04, *p* = 0.007; Figure 2B) and methylphenidate (10 mg/kg, i.p.) (time: *F*_(8,80)_ = 2.51, *p* = 0.019; Figure 2C) caused a pronounced increase in the relative dopamine release in the mPFC irrespective of the mouse line (time: *Fs* > 2.59, *p* < 0.019 line: *Fs* < 0.10, *p* > 0.752; time x line: *Fs* < 0.39, *p* > 0.916). Results were essentially the same for norepinephrine (time: *Fs* > 9.08, *p* < 0.001; time x line: *Fs* < 0.64, *p* > 0.744; Figures 2B,C), except for a significantly higher release in HAB mice following methylphenidate treatment (line: *F*_(1,77)_ = 5.43, *p* = 0.023). In each case, catecholamine levels peaked within the first 20 min after treatment, followed by a return towards basal levels within 2 h.

In the second cohort of mice (Experiment 2.2) microdialysis probes were implanted into the dorso-lateral part of the striatum (Figure 2D). Basal levels of dopamine were lower in LAB (*n* = 8) mice in comparison to HAB (*n* = 5) mice (*t*_(37)_ = 2.92, *p* = 0.006; Figure 2D). As shown by two-way ANOVAs, both amphetamine (1 mg/kg, i.p.) (time: *F*_(8,110)_ = 30.38, *p* < 0.001; Figure 2E) and methylphenidate (10 mg/kg, i.p.) (time: *F*_(8,80)_ = 43.21, *p* < 0.0001; Figure 2E) caused a significant increase in dopamine release, which was the same in HAB and LAB mice (line: *Fs* < 0.263, *p* > 0.668; time x line: *Fs* < 1.11, *p* > 0.365). Norepinephrine levels were below the detection limit. Again, dopamine levels peaked within 20 min after injection and returned to baseline within 2 h.

In a separate cohort of LAB and HAB mice we monitored changes in the distance traveled within 60–120 min after amphetamine (1 mg/kg, i.p.) and methylphenidate (10 mg/kg, i.p.) administrations (Experiment 2.3). It is of note that the transient increase in locomotor activity upon amphetamine (two-way ANOVA, treatment: *F*_(1,120)_ = 9.17, *p* = 0.0066; Figure 2F) and methylphenidate (two-way ANOVA, treatment: *F*_(1,33)_ = 47.11, *p* < 0.0001; Figure 2G) treatment in HAB and methylphenidate treatment in LAB mice (two-way ANOVA: treatment: *F*_(1,45)_ = 40.06, *p* = 0.0008; Figure 2G), with return towards basal levels within 1 h, closely resembled the release patterns of dopamine and norepinephrine. The calming effects of amphetamine in LAB mice (two-way ANOVA: treatment: *F*_(1,66)_ = 58.75, *p* < 0.0001; Figure 2F), in contrast, outlasted by far the neurochemical changes.

Collectively, the microdialysis experiments did not reveal any differences in dopamine or norepinephrine release upon treatment with amphetamine between LAB and HAB mice, which would explain the opposite behavioral effects. Moreover, amphetamine and methylphenidate showed comparable effects on dopamine and norepinephrine release in LAB mice despite their different effects on locomotor activity.

### Dopamine D2 Receptor Function is not Impaired in LAB Mice

There is evidence for a critical involvement of dopamine D2 receptor in amphetamine behavioral effects (Seeman and Madras, 1998; Beaulieu et al., 2004, 2005). Therefore, we investigated whether dopamine D2 receptor signaling was altered in LAB mice under basal conditions. To this end, we examined behavioral (locomotor activity and catalepsy) and neurochemical changes induced by systemic administration of haloperidol. Haloperidol (1 mg/kg, i.p.) equally decreased locomotor activity in the OF test in both LAB and HAB mice (Experiment 3.1) (two-way ANOVA, line: *F*_(1,27)_ = 1.75, *p* = 0.197; treatment: *F*_(1,27)_ = 63.48, *p* < 0.0001; line x treatment: *F*_(1,27)_ = 2.77, *p* = 0.109; Figure 3A); for details concerning the dynamics of locomotor activity changes see Yen et al. (2013). Haloperidol also caused a significant increase in catalepsy in LAB mice (Experiment 3.2). The descent latency from an involuntary posture increased 60 min after haloperidol administration, whereas amphetamine treatment (1 mg/kg, i.p.) had no effects compared to vehicle (one-way ANOVA: *F*_(2)_ = 10.84, *p* = 0.0004; Figure 3B). This proves the specificity of amphetamine-induced decrease in locomotor activity in these animals.

We also examined the efficacy of dopamine D2 receptors to affect dopamine release on the local level (presynaptic autoreceptor-mediated release regulation) (Experiment 3.3). As revealed by two-way ANOVA, haloperidol elicited an increase in dopamine (time: *F*_(11,93)_ = 2.32, *p* = 0.017; Figure 3C) and HVA (time: *F*_(11,80)_ = 28.49, *p* < 0.001; Figure 3D) levels in the mPFC, with no differences between the two lines (line: *Fs* < 2.41, *p* > 0.125, time x line: *Fs* < 1.46, *p* > 0.337).

Taken together, these results demonstrate intact functions of dopamine D2 receptors, both pre- and postsynaptic, in LAB mice. Moreover, the calming effect of amphetamine does not result from any cataleptic-like effect.

### GSK3β Inhibitors Attenuate Hyperlocomotion in LAB Mice

We showed before, that LiCl decreased the hyperactivity in LAB mice (Yen et al., 2013). Pharmacological activity of lithium is mediated by both direct and indirect inhibition of GSK3β (Beaulieu et al., 2009). Therefore, we compared the effects of amphetamine (1 mg/kg, i.p.), LiCl (100 mg/kg, i.p.), and the selective GSK3β inhibitor TDZD-8 (20 mg/kg, i.p.) on the locomotor activity in LAB, NAB, and HAB mice (Experiment 4.1 and 4.2). In LAB mice, TDZD-8 did not affect the locomotion in the OF test at a dose of 10 mg/kg, i.p. (data not shown). However, a dose of 20 mg/kg, i.p., sufficed to decrease hyperactivity. Notably, TDZD-8 elicited changes in locomotor activity which were qualitatively and quantitatively similar to the effects of amphetamine and LiCl: a rapid and lasting decline in activity (two-way ANOVA: treatment: *F*_(2,275)_ = 0.48, *p* = 0.625; time: *F*_(15,275)_ = 82.81, *p* < 0.0001; time x treatment: *F*_(15,275)_ = 2.47, *p* < 0.0001; Figure 4A). The increase in the selectivity of the GSK3β inhibitors (LiCl vs. TDZD-8) went along with a loss in line specificity in their calming action: Though LiCl decreased locomotor activity in NAB, but not in HAB mice, TDZD-8 was effective in all three lines. Two-way ANOVA (line x treatment) showed significance for each factor and their interaction (treatment: *F*_(1,61)_ = 21.685, *p* < 0.0001; line: *F*_(1,61)_ = 17.65, *p* < 0.0001; treatment x line: *F*_(1,61)_ = 7.44, *p* < 0.0001) (Figure 4B).

Although the acute effects of amphetamine and TDZD-8 were comparable in LAB mice, we observed a critical difference in delayed toxic impact of the two drugs: 2–3 days (but not 24 h) after treatment with TDZD-8 the animals showed a mortality rate of 30%. This may be related to a strong peripheral metabolic effect of the selective GSK3β inhibitor. In contrast, we failed to observe any fatal consequences for amphetamine treatment despite the huge number of treated mice (more than 100 over 3 years).

Similar profiles of hyperactivity mitigation after GSK3β inhibitor (TDZD-8, LiCl) and amphetamine administration in LAB mice strongly suggest that these compounds share a common molecular mechanism of action.

### Amphetamine Selectively Increases GSK3β Phosphorylation in the mPFC of LAB Mice

The similarities in the behavioral consequences of amphetamine and GSK3β inhibitors prompted us to investigate whether amphetamine directly interferes with GSK3β. To address this question, we examined the GSK3β inhibitory activity for amphetamine and methylphenidate *in vitro* (Experiment 5). Amphetamine and methylphenidate did not decrease the activity of purified GSK3β *in vitro*. In contrast, in this preparation, lithium effectively inhibited GSK3β, as well as did TDZD-8 (Figure 4C).

We next looked for changes in GSK3β phosphorylation *in vivo*, assuming an indirect interaction (Experiment 6). To this end, we treated HAB, NAB and LAB mice with amphetamine (1 mg/kg, i.p.) and measured the levels of phospho-Ser9-GSK3β (pGSK3β) in the mPFC (Figure 4D) and in the striatum (Figure 4E) 60 min later. Between-line (LAB, NAB, and HAB mice) comparison (two-way ANOVA, line, structure) revealed no difference in the phosphorylation for GSK3β, neither in the mPFC and in the striatum of vehicle-treated animals (line: *F*_(2,59)_ = 3.69, *p* = 0.307; structure: *F*_(1,59)_ = 4.21, *p* = 0.102; interaction: *F*_(1,59)_ = 2.21, *p* = 0.489; Figure 4F) nor in total GSK3β levels (not shown). This speaks against a significant role of GSK3β in basal differences in locomotor activity. Following amphetamine treatment of LAB mice, however, GSK3β phosphorylation was significantly increased in both the mPFC and striatum. Three-way ANOVA (line x structure x treatment) showed significance of each factor and their interactions (*Fs* > 6.62, *ps* < 0.0011). Respectively, the two-way ANOVA confirmed the effect of amphetamine in each line group separately (treatment: *F*_(1,33)_ = 47.58, *p* < 0.0001; structure: *F*_(1,33)_ = 0.57, *p* = 0.54; treatment x structure: *F*_(1,33)_ = 0.57, *p* = 0.54; Figures 4G,H). Amphetamine also increased the phosphorylation of GSK3β in NAB mice. This, however, was only the case for the striatum, but not for the mPFC (treatment: *F*_(1,26)_ = 21.44, *p* = 0.0001; structure: *F*_(1,26)_ = 24.22, *p* < 0.0001; treatment x structure: *F*_(1,26)_ = 24.22, *p* < 0.0001; Figures 4I,J). In HAB mice, we did not observe any changes in GSK3β phosphorylation, irrespective of the brain structure (treatment: *F*_(1,36)_ = 4.00, *p* = 0.217; structure: *F*_(1,36)_ = 2.21, *p* = 0.357; treatment x structure: *F*_(1,36)_ = 2.21, *p* = 0.357; Figures 4K,L).

In summary, amphetamine treatment caused line-dependent and structure-specific changes in GSK3β phosphorylation, whereby LAB mice displayed a selective increase in GSK3β phosphorylation in the mPFC.

### Selective Inhibition of GSK3β and Amphetamine Activity in the mPFC, but not in the Striatum, Mitigates Hyperlocomotion in LAB Mice

The Western blot data suggest a scenario according to which amphetamine mediates hypolocomotion via increased phosphorylation (i.e., inhibition) of GSK3β at the PFC rather than the striatal level. We verified this assumption by local application of amphetamine or TDZD-8 in the mPFC or striatum of LAB mice (Experiment 7) (Figures 5A,D). Bilateral administration of amphetamine (1.8 ng/0.5 ul/side) in the mPFC indeed decreased locomotor activity compared to vehicle immediately after drug infusion (two-way ANOVA: treatment: *F*_(1,165)_ = 2.00, *p* = 0.177; time: *F*_(11,165)_ = 8.66, *p* < 0.0001; treatment x time: *F*_(11,165)_ = 2.27, *p* = 0.013; Figure 5B). The same rapid effect was seen after TDZD-8 (1.1 ng/0.5 ul/side; treatment: *F*_(1,132)_ = 0.51, *p* = 0.488; time: *F*_(11,132)_ = 9.42, *p* < 0.0001; treatment x time: *F*_(11,132)_ = 3.38, *p* = 0.0004; Figure 5C). Bilateral administration of amphetamine (1.8 ng/0.5 ul/side) in the striatum, in contrast, rather stimulated locomotor activity (treatment: *F*_(1,66)_ = 0.71, *p* = 0.431; time: *F*_(11,66)_ = 6.68, *p* < 0.0001; treatment x time: *F*_(11,66)_ = 1.90, *p* = 0.054; Figure 5E), and TDZD-8 (1.1 ng/0.5 ul/side) had no effect at all (treatment: *F*_(1,132)_ = 0.04, *p* = 0.843; time: *F*_(11,132)_ = 9.72, *p* < 0.0001; treatment x time: *F*_(11,132)_ = 0.54, *p* = 0.870; Figure 5F).

These observations support the idea that the mPFC as a part of the activity controlling system (Carlsson and Carlsson, 1989) is involved in the paradoxical calming effect of amphetamine in LAB mice.

### The Action of Amphetamine in LAB Mice is Sensitive to NMDA Receptor Signaling in a GSK3β-Dependent Manner

Since dopamine and norepinephrine are unlikely to be causally involved in the calming effect of amphetamine at the level of the mPFC (Figure 2), we studied possible interactions between amphetamine and glutamate signaling. Previous studies have suggested such a possibility (Anderzhanova et al., 2001). To this end, we used a combined treatment of amphetamine and NMDA receptor blocker MK-801.

In the Experiment 8.1 MK-801 (0.3 mg/kg) injected alone increased locomotor activity both in HAB and LAB mice. Two-way ANOVA (time, treatment) of changes in absolute levels of the distance traveled showed in HAB mice significance of treatment: *F*_(1,180)_ 31.13, *p* = 0.0008; time *F*_(15,180)_ = 21.22, *p* < 0.0001; treatment x time: *F*_(15,180)_ = 15.31, *p* < 0.0001. In LAB mice two-way ANOVA revealed treatment: *F*_(1,165)_ = 35.02, *p* = 0.0001; time *F*_(15,165)_ = 10.71, *p* < 0.0001; treatment x time: *F*_(15,165)_ = 23.02, *p* < 0.0001; Figure 6A inset). Since the effect of the drug can be hindered by the difference in the basal activity we also analyzed relative changes in the locomotion under MK-801 treatment. The analysis of normalized data confirmed the elevation in locomotor activity by MK-801 both in HAB mice (two-way ANOVA, treatment: *F*_(1,180)_ = 236.0, *p* < 0.0001; time *F*_(15,180)_ = 14.20, *p* < 0.0001; treatment x time: *F*_(15,180)_ = 23.97, *p* = 0.0004) and LAB mice (treatment: *F*_(1,165)_ = 3.83, *p* = 0.076; time *F*_(15,165)_ = 3.58, *p* < 0.001; treatment x time: *F*_(15,165)_ = 9.37, *p* < 0.0001). Relative to the basal levels, MK-801 induced less pronounced elevation in the locomotor activity in LAB mice in comparison to HAB mice (two-way ANOVA: line: *F*_(1,165)_ = 14.10, *p* = 0.003; time: *F*_(15,165)_ = 17.87, *p* < 0.0001; line x time: *F*_(15,165)_ = 3.03, *p* < 0.0001; Figure 6A). This difference cannot be ascribed to divergence in habituation to the OF. When saline is injected, HAB, but not LAB, mice show a persistent decrease in locomotor activity (two-way ANOVA, line: *F*_(1,185)_ = 33.97, *p* < 0.0001; time: *F*_(15,165)_ = 24.30, *p* < 0.001; line x time: *F*_(15,165)_ = 7.38, *p* < 0.0001). In the Experiment 8.2 by microdialysis means we have shown that pretreatment (−20 min) with MK-801 (0.3 mg/kg, i.p.) did not facilitate amphetamine-induced dopamine release in the mPFC of LAB mice, whereas stimulated release in HAB mice (two-way ANOVA, line: *F*_(1,24)_ = 3.27, *p* = 0.048; time: *F*_(8,24)_ = 1.31, *p* = 0.28; line x time: *F*_(8,27)_ = 1.16, *p* = 0.32; Figure 6B, right panel). (The apparent decrease in dopamine release in LAB mice after MK-801 pretreatment compared to amphetamine given alone failed to reach the level of statistical significance).

In the distinct cohort of LAB mice (Experiment 9.1) MK-801 (0.3 mg/kg) pretreatment (−20 min) restored the hyperlocomotion in amphetamine-treated (1 mg/kg, i.p.) LAB mice (two-way ANOVA: treatment: *F*_(1,165)_ = 9.09, *p* = 0.0032; time: *F*_(15,165)_ = 12.22, *p* < 0.0001; treatment x time: *F*_(15,165)_ = 8.25, *p* < 0.0001; Figure 7A). MK-801 administered at the same time with amphetamine (+0 min) abolished the amphetamine calming effect (two-way ANOVA: treatment: *F*_(1,265)_ = 18.63, *p* = 0.0004, time: *F*_(15,265)_ = 30.35, *p* < 0.0001; treatment x time: *F*_(15,265)_ = 6.33, *p* < 0.0001; Figure 7B). Post-treatment (+20 min) with MK-801 (0.3 mg/kg, i.p.) in amphetamine- (1 mg/kg, i.p.) treated LAB mice, however, failed to interfere with its calming effect (two-way ANOVA: treatment: *F*_(1,180)_ = 4.10, *p* = 0.166; time: *F*_(1,15)_ = 42.74, *p* < 0.0001; treatment x time: *F*_(1,180)_ = 1.87, *p* = 0.722; Figure 7C).

Amphetamine (1 mg/kg) and MK-801 (0.3 mg/kg, i.p.) co-treatment resulted in a decrease in amphetamine-driven changes in phosphorylation of GSK3β in the mPFC 60 min after injection (Experiment 9.2) (*t*_(8)_= 3.12, *R*^2^= 0.564, *p* = 0.012; Figures 7D,E).

MK-801 (0.3 mk/kg, i.p.) pre-treatment prevented the calming effect of non-selective GSK3β inhibitor LiCl (100 mg/kg, i.p.) in LAB mice (Experiment 9.3) (two-way ANOVA: treatment: *F*_(1,150)_ = 8.10, *p* = 0.057; time: *F*_(1,15)_ = 12.21, *p* < 0.0001; treatment x time: *F*_(1,150_) = 16.30, *p* < 0.0001; Figure 7F). In contrast, MK-801 (0.3 mg/kg, i.p.) co-administered with the selective GSK3β inhibitor TDZD-8 (20 mg/kg, i.p.) (Experiment 9.4) failed to prevent its calming effect (two-way ANOVA, treatment x time: treatment: *F*_(1,270)_ = 0.264, *p* = 0.614; time: *F*_(15,270)_ = 79.00, *p* < 0.0001; treatment x time: *F*_(15,270)_ = 7.005, *p* < 0.0001; Figure 7G. It is know that in addition to direct inhibition of GSK3β activity lithium interacts with upstream factors of GSK3β phosphorylation. Therefore, MK-801 action likely targets upstream pathway(s) of GSK3β activity regulation.

Since both the dynamics and magnitude of hyperlocomotion mitigation in LAB mice were similar after systemic administration of amphetamine, and TDZD-8 (see Figure 4A), the differences in MK-801-amphetamine vs. MK-801-TDZD-8 interaction cannot be explained by insufficiency of amphetamine. Taken together, the behavioral and molecular data suggest an interaction of amphetamine and NMDA receptor signaling upstream of GSK3β activity in mediating amphetamine calming effect.

## Discussion

We examined the neurochemical and molecular signature of the amphetamine calming effect in LAB mice, which are characterized by the locomotor hyperactivity and cognitive impairment resembling an ADHD-like endophenotype (Yen et al., 2013). Our findings suggest that changes in dopamine and norepinephrine release in the mPFC and the striatum in LAB mice are unlikely involved in the calming action of amphetamine. Instead, we provide evidence that amphetamine actions involve inhibition of GSK3β at the level of the mPFC and interaction with NMDA receptor signaling.

We employed a line comparison strategy to identify the signature of the calming amphetamine effect in LAB mice. A set of behavioral and microdialysis experiments render it highly unlikely that changes in monoamine release play a major role in the line-specific hyperactivity and the calming effect of amphetamine: First, LAB mice showed lower, but not higher basal dopamine levels in the striatum (Figure 2D) compared to HAB mice. This not only corroborates findings in spontaneously hypertensive hyperactive rats (Russell et al., 1998; Russell, 2002), but speaks against the hypothesis that high basal dopamine levels are causally involved in ADHD-like hyperactivity (Waldman et al., 1998; Gainetdinov et al., 1999).

Second, both amphetamine and methylphenidate cause a similar increase in the dopamine and/or norepinephrine levels in LAB and HAB mice despite the line-specific difference in behavior (increased locomotion in HAB vs. decreased locomotion in LAB mice after amphetamine treatment compared to increased locomotion in both lines after methylphenidate treatment). The similar dynamics of neurochemical and behavioral changes after methylphenidate are consonant with an involvement of increased dopamine and/or norepinephrine signaling in the increased locomotion observed in both LAB and HAB mice. The divergent neurochemical (transient increase) and behavioral (sustained decrease) changes after amphetamine treatment in LAB mice, in contrast, argue against a causal relationship between an amphetamine-induced monoamine release and a paradoxical calming effect (Figures 1, 2). There are few reports of methylphenidate ineffectiveness (30–40%) in treatment of hyperactivity in kids (Winsberg et al., 1980; Pelham et al., 1999; Gerwe et al., 2009). At the same time amphetamine effective in a majority of cases (90%) (Pliszka et al., 2000). Therefore, differential response to amphetamine and methylphenidate in clinic may serve for a better diagnosis either to differentiate co-morbid disorders or the ADHD presentations.

Third, compared to HAB mice, MK-801 pre-treatment failed to facilitate amphetamine-induced dopamine release in the mPFC of these animals, but at the same time prevented from changes in the locomotor activity in LAB mice (Figures 6B, 7A,B). This serves as an additional proof of dopamine-independent hyperactivity mitigation in LAB mice and points on possible changes in the NMDA receptor-dependent activity of the mPFC.

Forth, neuroleptic haloperidol, the dopamine D2 receptor antagonist, decreased locomotion in LAB, NAB, and HAB mice to the same extent, induced catalepsy in LAB mice (in contrast to amphetamine), and elicited comparable changes in the dopamine and HVA levels in the mPFC of LAB and HAB mice (Figure 3), suggesting unaltered D2 receptor signaling in LAB mice. Finally, the lack of line differences in phosphorylation of GSK3β between saline-treated LAB, NAB, and HAB mice (Figure 4F) points towards intact regulation of the β-arrestin2/Akt/GSK3β complex at basal conditions (Beaulieu et al., 2005), and undisturbed dopamine D2 receptor-mediated signaling in LAB mice.

Mitigation of hyperactivity by amphetamine in LAB mice coincided with an increase in GSK3β phosphorylation both in the mPFC and in the striatum (Figures 4G,H). This effect was line-specific only at a level of the mPFC in which neither NAB nor HAB mice showed similar changes. This suggested that the inhibition of GSK3β activity in the mPFC contributes to the calming effects of amphetamine. In mice with normal locomotor activity, an increase in the brain GSK3β phosphorylation in the striatum and the mPFC may be observed as early as 15 min after amphetamine given systemically, whereas GSK3β phosphorylation is decreased in the striatum 60–90 min after amphetamine administration (Svenningsson et al., 2003; Beaulieu et al., 2005). However, Akt-independent increase in GSK3β phosphorylation in the mPFC was shown after recurrent amphetamine administration in a model of amphetamine-induced psychomotor sensitization. This phenomenon may involve activation of a short-cut GSK3β–β-arrestin2 feedback (Mines and Jope, 2012).

The short-term mitigation of hyperactivity resulting from local microinjections of amphetamine and TDZD-8 in the mPFC, but not in the striatum of LAB mice (Figure 5) supports the role of mPFC in the observed pharmacological phenomenon. In order to avoid drugs being spread across the structures, we applied low doses that probably resulted in the rapid and non-lasting drug effect. The transient effect of microinjections compared to the results of systemic treatment can be also explained considering the difference in the pharmacokinetics of drugs at local and i.p. administration.

Currently, we can only speculate about the way how the selective decrease in GSK3β activity in the mPFC is translated into behavior. Since the majority of mPFC neurons (70–75%) are glutamatergic neurons, it is tempting to assume that the prominent molecular changes are mediated by alterations in the projecting glutamatergic neurons of the mPFC. An alternative scenario is taking into account the differences in pharmacodynamic aspects of amphetamine and methylphenidate action (Calipari et al., 2015). In contrast to methylphenidate, amphetamine targets the DAT and other monoamine transporters, pumps the neurotransmitters out of the terminal, but does not block their uptake. Amphetamine modulates the phasic release of dopamine, inhibits vesicular monoamine transporter type 2 and monoamine oxidase. Its metabolites may also contribute to the profile of amphetamine actions (Sulzer, 2011). In addition, amphetamine changes the performance of amino acid transporters (Del Arco et al., 1999), which results in an increase in the extracellular glutamate levels (Anderzhanova et al., 2001). As has been recently reported, LAB and HAB mice are strictly different in the expression of proteins involved in the regulation of the glutamatergic and GABAergic neurotransmission at the level of the cingulate cortices (Filiou et al., 2011; Iris et al., 2014). Moreover, a lower plasma level of glutamate was found in LAB mice (Zhang et al., 2011). Together, these observations support the idea of a possible imbalance of the excitatory and inhibitory neurotransmission in LAB mice in general. Interestingly, a disturbance in the glutamatergic neurotransmission was proposed as a mechanism mediating hyperactivity in DAT-KO mice (Gainetdinov et al., 2001).

Given the fact that amphetamine may cause glutamate release, our observations provide a mechanistic explanation for the specific amphetamine action in LAB mice. The relatively small increase in locomotor activity in LAB mice after MK-801 systemic administration in comparison to HAB mice (Figure 6A) and the lack of its facilitating effect on amphetamine-evoked dopamine release in the mPFC (Figure 6B) point to innate changes in NMDA receptor-mediated activity in these animals (Duncan et al., 2002). The interpretation of our behavioral data on MK-801 activity may be limited due to difference in the basal locomotor activity between LAB and HAB mice. In fact, the normalization algorithm we applied to compare MK-801 effect between lines may potentially lead to the drug effect overestimation. Thus, original data show that LAB mice develop higher absolute locomotor activity after MK-801 administration than HAB mice. Nonetheless, a summation of the basal activity and MK-801 induced effect may be achieved in LAB mice due to the same function (constitutional or antagonist-induced decrease in NMDA receptor activity). Our consideration that the basal hyperlocomotion and diminished relative effect of MK-801 in LAB mice have same nature is supported by our neurochemical and molecular data. A possible hypofunctionality of NMDA receptors in GABA-ergic cortical neurons (Corlett et al., 2011) may underlie psychotic traits of the endophenotype representing an ADHD-mania-schizophrenia continuum (Yen et al., 2013). Our findings of differentially timed MK-801 and amphetamine treatment (Figures 7A,B,C) and lack of TDZD-8 and amphetamine interaction (Figure 7G) suggest that amphetamine directs its action at GSK3β in the mPFC via a pathway, which depends on upstream NMDA receptor-mediated quasi-metabotropic signaling (Figure 7H). This NMDA receptor-mediated GSK3β activity regulation may be independent of pathways forcing Akt phosphorylation at the Ser308. Preliminary data show that the Thr473 phosphorylation site is rather engaged, since we have observed a line x structure-dependent correlation between changes in the levels of phospho-Thr473-Akt and phospho-Ser9-GSK3β under amphetamine treatment.

In conclusion, neither the hyperactivity in LAB mice nor the calming effect of amphetamine can be ascribed to changes in dopamine and norepinephrine neurotransmission in the striatum and the mPFC. Instead, amphetamine-triggered phosphorylation of GSK3β in the mPFC, but not the striatum, seems to participate in amphetamine-induced mitigation of hyperactivity in LAB mice. This calming action of amphetamine involves a functional interaction with NMDA receptors upstream of GSK3β. From a translational perspective, our data suggest GSK3β as a target for pharmacotherapy of disorders from the ADHD-mania-schizophrenia continuum.

## Author Contribution

Y-CY, AZ, NCG, EA acquired data; NCG, TR, EA, CTW designed the work; EA, RL, CTW conceived the work and played an important role in interpreting the results; EA drafted the manuscript; EA and CTW contributed equally to the study.

## Conflict of Interest Statement

The authors declare that the research was conducted in the absence of any commercial or financial relationships that could be construed as a potential conflict of interest. This work was supported by the Max Planck Society.
